# Quantitative and qualitative characterization of expanded CD4^+^ T cell clones in rheumatoid arthritis patients

**DOI:** 10.1038/srep12937

**Published:** 2015-08-06

**Authors:** Kazuyoshi Ishigaki, Hirofumi Shoda, Yuta Kochi, Tetsuro Yasui, Yuho Kadono, Sakae Tanaka, Keishi Fujio, Kazuhiko Yamamoto

**Affiliations:** 1 Department of Allergy and Rheumatology, Graduate School of Medicine, the University of Tokyo, Tokyo, Japan; 2 Laboratory for Autoimmune Diseases, Center for Integrative Medical Sciences, RIKEN, Yokohama, Japan; 3 Department of Orthopaedic Surgery, Graduate School of Medicine, the University of Tokyo, Tokyo, Japan

## Abstract

Rheumatoid arthritis (RA) is an autoimmune destructive arthritis associated with CD4^+^ T cell-mediated immunity. Although expanded CD4^+^ T cell clones (ECs) has already been confirmed, the detailed characteristics of ECs have not been elucidated in RA. Using combination of a single-cell analysis and next-generation sequencing (NGS) in TCR repertoire analysis, we here revealed the detailed nature of ECs by examining peripheral blood (PB) from 5 RA patients and synovium from 1 RA patient. When we intensively investigated the single-cell transcriptome of the most expanded clones in memory CD4^+^ T cells (memory-mECs) in RA-PB, senescence-related transcripts were up-regulated, indicating circulating ECs were constantly stimulated. Tracking of the transcriptome shift within the same memory-mECs between PB and the synovium revealed the augmentations in senescence-related gene expression and the up-regulation of synovium-homing chemokine receptors in the synovium. Our in-depth characterization of ECs in RA successfully demonstrated the presence of the specific immunological selection pressure, which determines the phenotype of ECs. Moreover, transcriptome tracking added novel aspects to the underlying sequential immune processes. Our approach may provide new insights into the pathophysiology of RA.

Rheumatoid arthritis (RA) is an autoimmune disease that is characterized by systemic chronic synovitis and bone erosion. Although various types of innate and adaptive immune cells have been shown to coordinately contribute to the pathophysiology of RA, evidence obtained from human^1^,^2^,^3^ and mouse studies^4^,^5^,^6^,^7^ has suggested the central roles of CD4^+^ T cells. Clonal expansion is a unique feature of the adaptive immune system, which makes it efficient and potent for antigen-specific reactions. A detailed characterization of clonal expansion is informative for assessing abnormalities in autoimmune diseases, especially antigen-specific autoimmunity. T cell receptor (TCR) repertoire analysis is the standard approach used to assess the clonal expansion status and many studies have focused on expanded CD4^+^ T cell clones (ECs) in RA^8^,^9^,^10^. However, only limited fraction of CD4^+^ T cells were analyzed in previous articles and it was methodologically impossible to characterize the phenotypes of ECs.

Complementarity-determining region 3 (CDR3) of TCR has theoretically 10^15^ diversity^11^ and can be used as a unique marker of each T cell clone. Historically, single strand conformation polymorphism (SSCP) and Sanger’s sequencing have been used for TCR analyses^9^,^12^,^13^. Recent advances in next-generation sequencing (NGS) has enabled us to perform quantitative analyses of the TCR repertoire with large amounts of TCR sequencing data. Although the NGS TCR repertoire analysis is becoming a widely accepted method^14^,^15^,^16^, it can be affected by PCR bias and has a higher error rate than that of classical Sanger’s sequencing^17^. Sequencing error is a critical issue especially in TCR sequencing. Random V-D-J recombination makes the diversity of CDR3 so enormous that it is difficult to distinguish sequencing errors from rare clones. Although there have been some studies on error correction algorithms for NGS TCR repertoire analysis^18^, careful applications are required because inaccurate algorithm might distort the results of TCR repertoire analysis. On the other hand, TCR repertoire analysis based on single-cell Sanger’s sequencing has complementary features to NGS: negligible PCR bias and a low error rate. Therefore, the accuracy and the validity of error correction algorithms for NGS TCR repertoire analysis have to be tested using reference data obtained by single-cell Sanger’s sequencing. However, few studies to date have directly compared NGS and single-cell TCR repertoire analyses.

Due to the lack of reliable markers of clonal expansion, single-cell transcriptome analysis is the only method that allows ECs to be characterized because TCR sequencing and gene expression analysis have to be investigated simultaneously and analyzed comprehensively in a single-cell resolution. The extremely small amount of total RNA (approximately 10 pg total RNA/single cells) has been the main obstacle to single-cell gene expression analysis. However, recent improvements in experimental methodology have made single-cell transcriptome analysis feasible by efficient amplifications of single-cell cDNA and the suppression of PCR byproducts^19^,^20^,^21^.

The combination of a comprehensive TCR repertoire analysis and single-cell transcriptome analysis now allows ECs to be characterized quantitatively and qualitatively. Moreover, single-cell transcriptome analysis also enables us to track the gene expression changes of the same clones in different sites. In this way, through in-depth characterization of ECs, we might be able to detect pathogenic clones and unveil the new aspects of pathophysiology of RA.

In the present study, we began our experiments by validating the NGS TCR repertoire analysis using a single-cell analysis. The TCR repertoire of different subsets of CD4^+^ T cells in peripheral blood (PB) from 5 RA patients and 5 healthy controls was then analyzed by NGS. We finally performed a single-cell transcriptome analysis of the most expanded CD4^+^ T cell clones (mECs) in PB and the synovium and tracked the shift in gene expression profiles between them.

## Results

### ECs were continuously detected in long-standing RA

**Figure 1:**
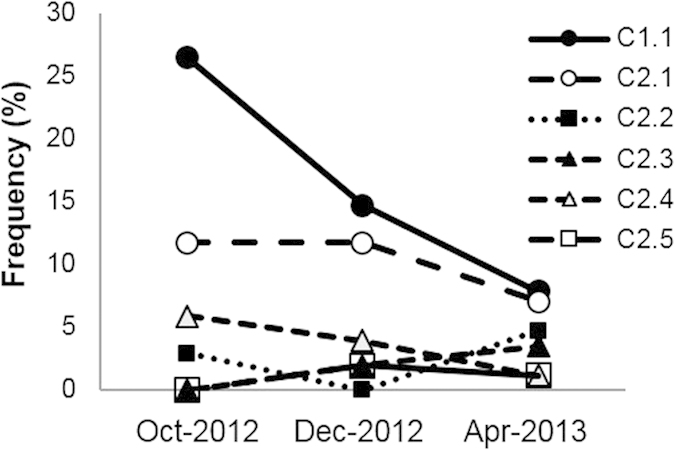
Major memory-ECs were repeatedly detected in RA-PB. Single-cell analysis of PB memory CD4^+^ T cells from RA1 and RA2 was repeatedly performed with 3-month intervals and the TCR repertoire was analyzed. Each dot and line indicates one clone. Ci.j is the clone ID indicating the j-th expanded major clone in RAi.

The existence of ECs has been reported previously in both healthy individuals and RA patients^22^,^23^. We first examined the persistence of ECs over several months. In two RA patients with stable disease and fixed treatments, the TCR repertoire analysis of PB memory CD4^+^ T cells was repeatedly performed with 3-month intervals by a single-cell analysis, which is the gold standard method. Since we sorted a maximum of 102 single cells per one sample, we defined ECs in the single-cell analysis as CD4^+^ T cell clones observed more than once. We detected several identical memory-phenotype ECs (memory-ECs) repeatedly from each patient and confirmed the persistent oligoclonal expansion of major memory-ECs in RA-PB (Fig. 1). Detailed information on the main memory-ECs detected in this pipeline was listed in Table S3. We showed that memory-ECs continuously occupied a significant percentage of memory CD4^+^ T cells and these results suggested the existence of chronic selection pressure.

### Naive and Memory CD4^+^ T cells had a distinct TCR repertoire

**Figure 2:**
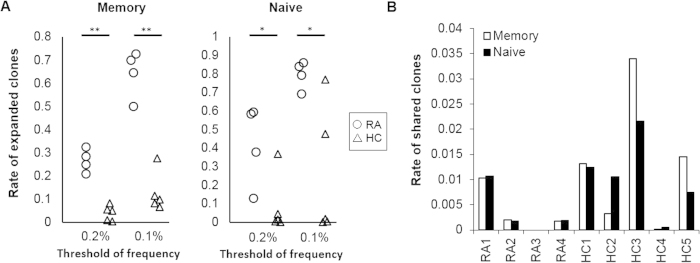
Both of naive and memory CD4^+^ T cells in RA-PB had many ECs. Naïve and memory CD4^+^ T cells were sorted from the PB of 4 RA patients and 5 healthy controls and the NGS TCR repertoire analysis was performed. (**A**) Rate of expanded clones (clones with more than 0.2% or 0.1% of total number of reads with functional CDR3 sequences). The cumulative read count of ECs was divided by the total number of reads with functional CDR3 sequences. (**B**) Rate of clones that were shared by both the naive and memory CD4^+^ T cell subset. The cumulative read count of shared clones was divided by the total number of reads with functional CDR3 sequences. *p < 0.05, **p < 0.005.

In order to characterize the TCR repertoire of CD4^+^ T cells in RA, we sorted naive and memory CD4^+^ T cells from RA-PB and healthy control (HC) PB and performed the NGS TCR repertoire analysis. We first verified the robustness of our NGS TCR repertoire analysis platform by single-cell analysis and FACS analysis (Fig. S1 and S2). We were able to identify ECs by high-frequency CDR3 sequences in the NGS TCR repertoire analysis. Since there is currently no general definition of ECs in the NGS TCR repertoire analysis, we prepared two thresholds of frequencies for ECs (Fig. 2A, 0.2% and 0.1%). We observed significantly more ECs in not only memory, but also naive CD4^+^ T cells in RA-PB than in healthy control PB. When the similarities of sequences between memory and naive CD4^+^ T cells were examined, we found that the majority of clones in each subset were unique and not shared by the other subset both in RA and HC (Fig. 2B). These results revealed marked differences in the TCR repertoire between naive and memory CD4^+^ T cells.

### The non-Th1/Th17/Tfh subset in RA-PB contained the majority of expanded and synovium-infiltrating clones

Memory CD4^+^ T cells are functionally heterogeneous and can be classified into subpopulations by characteristic cytokine productions, transcription factor networks and epigenomic changes^24^,^25^,^26^,^27^. Among the subpopulations of memory CD4^+^ T cells, Th1, Th17 and Tfh have been reported to have critical roles in the pathogenesis of RA and other autoimmune diseases^28^,^29^,^30^,^31^,^32^. In order to further characterize the TCR repertoire of memory CD4^+^ T cells in RA, we sorted Th1, Th17, Tfh, and non-Th1/Th17/Tfh subsets (see details in methods) and performed the NGS TCR repertoire analysis. Non-Th1/Th17/Tfh subsets contained Th2 and other subsets which are not well characterized in the current understanding of human immune system.

**Figure 3:**
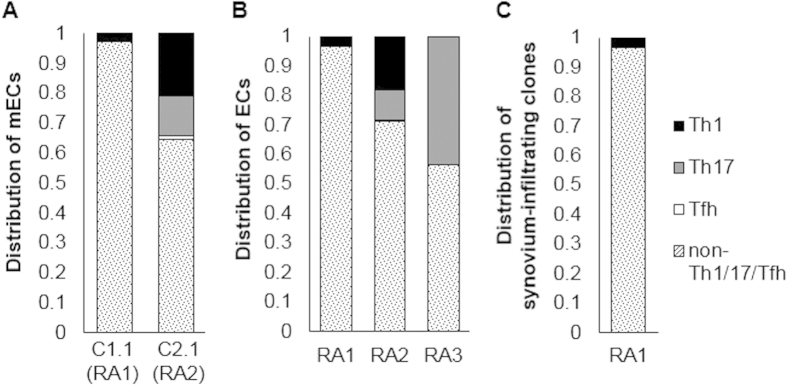
The non-Th1/Th17/Tfh subset in RA-PB contained the majority of expanded and synovium-infiltrating clones. The Th1, Th17, Tfh and non-Th1/Th17/Tfh subsets were sorted from 3 RA-PB and the NGS TCR repertoire analysis was performed. In order to assess the distribution of target clones within different subsets of CD4^+^ T cells, frequencies assessed by the TCR repertoire analysis (the total number of reads of target clones divided by the total number of reads with functional CDR3 sequences) were multiplied by the frequency of each CD4^+^ T cell subset within all CD4^+^ T cells assessed by FACS in order to correct for the subset size. (**A**) The distribution of the mECs in RA1 and RA2 (corresponding to C1.1, C2.1 in Fig. 1). (**B**) The distribution of ECs (clones with more than 0.2% of total number of reads with functional CDR3 sequences). (**C**) The distribution (within PB) of clones that were also detected in the synovium of the same patient.

We initially focused on the most expanded CD4^+^ T cell clones (mECs) that persistently expanded with the highest frequency as shown in Fig. 1 (C1.1 for RA1 and C2.1 for RA2). Both of these were the most frequently observed in the non-Th1/Th17/Tfh subsets (Fig. 3A). The distributions of target clones (Fig. 3A–C) were calculated based on the frequency assessed by the TCR repertoire analysis and the size of each subset assessed by FACS analysis (see details in the figure legend). We then examined the distribution of ECs (more than 0.2%) in the 4 subsets of memory CD4^+^ T cells and found that the majority of ECs were also detected in the non-Th1/Th17/Tfh subsets (Fig. 3B). We tracked synovial tissue-infiltrating CD4^+^ T cells and examined their localization in PB. A comparison of the PB and synovial tissue TCR repertoires from RA1 revealed that the non-Th1/Th17/Tfh subsets contained the majority of synovial tissue-infiltrating CD4^+^ T cells (Fig. 3C). The distinct clonality of ECs between naïve and memory CD4^+^ T cells and evident skewing of memory ECs toward the non-Th1/Th17/Tfh subsets collectively suggested that the expansion of ECs reflected a specific differentiation process rather than a non-specific generalized activation process.

### Gene expression profiles of mECs in RA-PB

**Figure 4:**
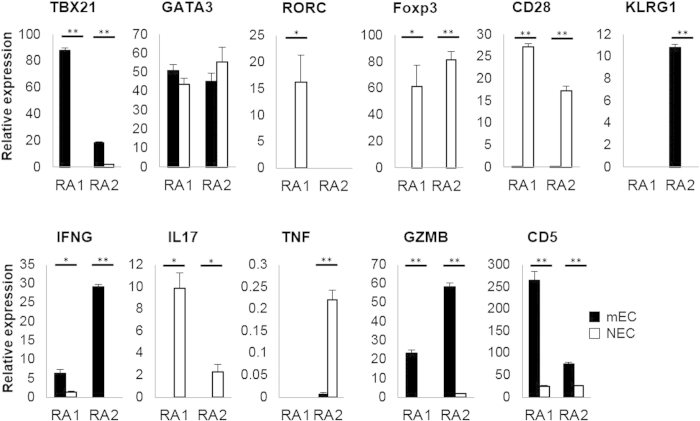
Gene expression profiles of PB memory-mECs. cDNAs of PB memory-mECs (corresponding to C1.1 for RA1 and C2.1 for RA2 as shown in Fig. 1) and memory-NECs obtained by the single-cell analysis were mixed (4 pooled samples in total), and qPCR was performed. ACTB was used as an internal control gene. *p < 0.05, **p < 0.005.

In order to further characterize the memory ECs in RA, we next performed single-cell transcriptome analysis of the most expanded CD4^+^ T cell clones (mECs) and non-expanded CD4^+^ T cell clones (NECs) in the peripheral blood of 2 patients, RA1 and RA2 (Fig. 4). We defined mECs and NECs as the CD4^+^ T cell clones detected in the single-cell analysis of each sample with the highest frequency and only once, respectively. We focused on “the most expanded” clones for this purpose because multiple single cells are required for the reliable characterization of the gene expression profile of each clone. We also confined our target to memory-phenotype CD4^+^ T cell clones (memory-mEC and memory-NECs). By comparing memory-mECs with memory-NECs from the same patient, we could extract the “clonal expansion” molecular signatures, without being influenced by inter-individual variability.

In order to capture the whole view of the gene expression characteristics of the memory-mECs, we first divided our single-cell cDNA samples into 4 groups: memory-mECs (8 cells) and memory-NECs (65 cells) in RA1 PB and memory-mECs (6 cells) and memory-NECs (48 cells) in RA2 PB. Due to a limitation in the cDNA sample volume, every single-cell cDNA of each group was mixed and quantitative PCR (qPCR) was performed for each pooled sample.

The down-regulation of CD28 and up-regulation of KLRG1 and GZMB were observed in memory-mECs, and this was similar to the phenotype of senescent CD4^+^ T cells^33^. T cell senescence is one of the characteristic findings in the immunological abnormalities of RA^34^,^35^. Memory-mECs also had a gene expression profile that was similar to Th1 (up-regulation of TBX21 and IFNG). The down-regulation of RORC, IL17, and Foxp3 suggested that memory-mECs in RA-PB did not belong to the Th17 or Treg subsets.

### Gene expression profiles of mECs in the RA synovium

**Figure 5:**
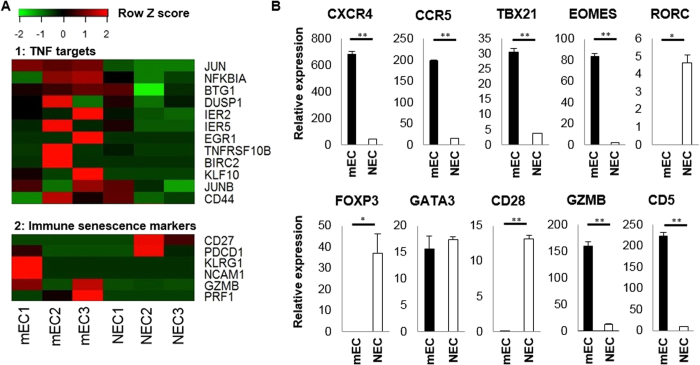
Gene expression profiles of synovium memory-mECs. Three single-cell cDNA samples were randomly selected from both memory-mECs and memory-NECs in the synovium, and analyzed by single-cell RNA-Seq. (**A)** A heatmap of TNF target genes and immune senescent markers. The TNF targets genes were shown to be significantly up-regulated in memory-mECs by the gene set enrichment analysis (see details in the text and supplementary methods). (**B)** cDNAs of synovium memory-mECs and memory-NECs obtained by the single-cell analysis were mixed (2 pooled samples in total), and qPCR was performed. ACTB was used as an internal control gene. *p < 0.05, **p < 0.005.

We next performed single-cell transcriptome analysis of synovium of RA1 (Fig. 5). Three single-cell cDNA samples were randomly selected from both synovium memory-mECs and memory-NECs, and analyzed using single-cell RNA-Seq. An average of 1.46 million reads was mapped per sample. In order to assess differentially expressed gene sets, we performed a gene set enrichment analysis^36^ and found that the expression of the gene sets of TNF targets were significantly higher in synovium memory-mECs than in synovium memory-NECs (FDR = 0.029. Heatmap was shown in Fig. 5A).

When gene expression was compared between the pooled cDNA of synovium memory-mECs (4 cells) and memory-NECs (15 cells), the significant up-regulation of CXCR4 was observed in memory-mECs (Fig. 5B). CXCR4 is an important chemokine receptor for homing to synovial tissues and was previously shown to be associated with the pathogenicity of arthritis^37^,^38^. The down-regulation of RORC and Foxp3 also suggested that memory-mECs in the synovium did not belong to the Th17 or Treg subsets, as in RA-PB. We collected immune senescence marker genes^39^,^40^ from RNA-seq data and summarized them in a heatmap (Fig. 5A). The heatmap and qPCR data collectively suggested that synovium memory-mECs also possessed a senescent CD4^+^ T cell-like gene expression profile (up-regulation of KLRG1, NCAM1, GZMB, and PRF1 and down-regulation of CD27, CD28, and PDCD1).

### Gene expression tracking of mECs

**Figure 6:**
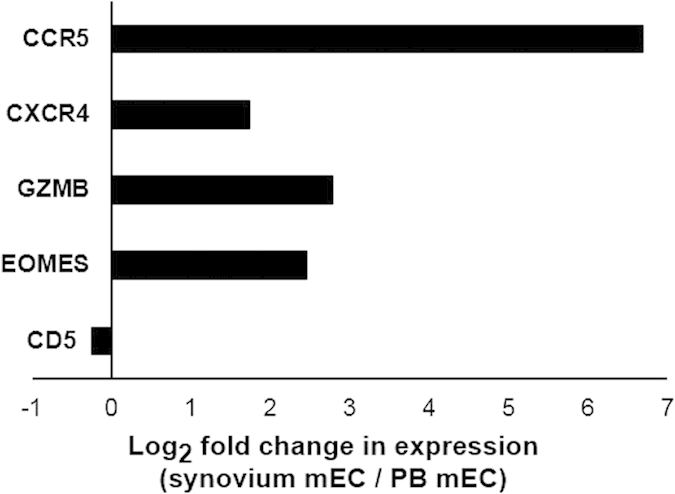
Tracking of the gene expression shift in memory-mECs in RA1 between PB and the synovium. Memory-mECs in the RA1 PB and RA1 synovium were identical clones (corresponding to C1.1 in Fig. 1). cDNAs of RA1 memory-mECs in PB and the synovium obtained by the single-cell analysis were mixed (2 pooled samples in total), and qPCR was performed. ACTB was used as an internal control gene. The log-transformed ratio (gene expression value in the synovium / gene expression value in PB) was shown.

The combined use of single-cell TCR repertoire analysis and single-cell transcriptome analysis enabled us to track the gene expression shift within the same clones by localization. Since memory-mECs in the RA1 PB and the RA1 synovium were identical clones (corresponding to C1.1 in Fig. 1), we examined the phenotypic changes of memory-mECs caused by infiltration into the synovium (Fig. 6). Chemokine receptors, which are important for infiltration into the synovium (CXCR4 and CCR5), and effector molecules (GZMB) were up-regulated in the synovium. On the other hand, CD5, a marker of autoreactivity^41^, was stably expressed.

## Discussion

In the present study, we proposed an original and rational strategy for the detailed characterization of each EC quantitatively (level of clonal expansion) and qualitatively (infiltration into target organs, cell surface markers, and gene expression status) by combining a comprehensive TCR repertoire analysis and single-cell transcriptome analysis. Although the clonal size and senescence features of ECs in RA-PB have already been reported^23^,^33^, the mode of proliferation of ECs has not yet been clarified. ECs may expand non-specifically among various kinds of T cell subpopulations including naïve and memory T cell subsets. We succeeded in adding novel aspects to the dynamic processes underlying memory-ECs in RA. The persistently high level of expansion of memory-ECs and apparent skewing of their phenotype to distinct subsets clearly demonstrated that ECs were constantly exposed to specific immunological selection pressure. Moreover, tracking of the gene expression profiles of memory-mECs suggested that they underwent sequential immunological modifications. Memory-mECs in PB acquire an immune senescence phenotype by chronic selection pressure and up-regulate the expression of chemokine receptors for homing to the synovium. Memory-mECs are exposed to TNF in the synovium and augment pathogenic activity. We speculated that this sequential phenomenon is not a unique event specific to memory-mECs, but a generalized process for other autoreactive and joint-homing memory CD4^+^ T cells.

Most of the results obtained in the present study are consistent with previous findings. Senescent CD4^+^ T cells were previously reported to exhibit high levels of clonal expansion and play pathogenic roles in RA through cytotoxic molecules such as perforin and granzymes^33^,^40^,^42^,^43^. TNF signaling appeared to function as a booster of senescence in synovium memory-mECs because TNF alpha was shown to augment the senescent phenotype in CD4^+^ T cells^44^,^45^. EOMES is one of the molecular signatures of senescence, although this finding came from CD8^+^ T cells^46^. The upregulation of this transcript in RA-PB memory-mECs and more prominently in synovium memory-mECs also appeared to be associated with the senescent phenotype.

Unlike Th1, Th17 and Tfh subsets, non-Th1/Th17/Tfh subset (Th2 and other subsets which are not well characterized) has not been considered and investigated as a pathogenic population in RA. Our findings suggested there might be a novel pathogenic subpopulation in this subset. Although the majority of mECs were detected in the non-Th1/Th17/Tfh subset, some of them were also detected in Th1 and they had the transcriptomes similar to those of the Th1 subset. These results can be explained by inter-subset plasticity or the instability of CXCR3 expression because the discrimination of non-Th1/Th17/Tfh and Th1 was solely dependent on CXCR3 in this study, according to a standard strategy^47^.

The critical question of what the cognate antigens of these ECs are remains. The strong expression of CD5 in memory-mECs, which has been associated with autoreactivity^41^, indicated that they were autoreactive clones. Recent studies identified citrullinated vimentin, aggrecan, fibrinogen and BiP as the candidate auto-antigens of CD4^+^ T cells in RA based on the autoantibody repertoire and other factors^48^,^49^,^50^,^51^. The MHC class II tetramer assay showed that the frequency of CD4^+^ T cells specific to citrullinated epitopes was approximately 0.1–10 cells per 1,000,000 T cells without *in vitro* antigen stimulation^2^,^29^. The clonal expansion size of ECs was more than one thousand times larger than that of citrullinated auto-antigen-specific CD4^+^ T cells. Therefore, we speculated that the ECs detected in the present study recognized autoantigens other than the above antigens.

Current treatment of RA suppresses inflammation nonspecifically and the risk of adverse events such as infection is inevitable. Although data is limited, several autoantigens have already been identified and there have been several reports about the tolerance-inducing antigen-specific strategies^52^,^53^,^54^,^55^,^56^,^57^. By expanding our knowledge of autoantigens in RA and tolerance-inducing mechanism in human, we may be able to develop antigen-specific therapies which have fewer side effects and possibly become alternative treatment.

The analyzed sample size was evidently not sufficient to generalize our results. Nevertheless, we consider our combined analysis of PB and the synovium in one patient to have reflected at least some common RA features because RA1 was a prototypical seropositive patient with shared-epitope HLA-DRB1 alleles (HLA-DRB1*04:05/0101, Table S1).

Information regarding the physiological or pathogenic roles of ECs is currently limited. The unbiased and robust nature of our strategy should shed light on these roles and may ultimately be able to identify a new aspect of RA pathophysiology.

## Methods

### Patients and controls

PB was obtained from 5 RA patients visiting outpatient clinics of the Department of Allergy and Rheumatology at The University of Tokyo Hospital and 5 healthy donors. Synovial tissue was collected from 1 active RA patient (harboring HLA-DRB1*04:05, positive for both RF and anti-CCP antibodies) during elbow synovectomy at the Department of Orthopaedic Surgery and Spinal Surgery. Detailed patient information is summarized in Table S1. RA was diagnosed according to the American College of Rheumatology/European League Against Rheumatism 2010 criteria^58^. This study was approved by the Ethical Committee of The University of Tokyo. A written form of informed consent was obtained from all patients and healthy donors. The methods were carried out in accordance with the approved guidelines.

### Cell isolation

PB mononuclear cells were separated by Ficoll-Hypaque density-gradient centrifugation^59^. Synovial lymphoid cells were also enriched by Ficoll-Hypaque from cell suspensions made by passing synovial tissue through a stainless steel sieve.

### Flow cytometry (FACS)

We analyzed and sorted all samples for single-cell and NGS analyses by MoFlo XDP (Beckman Coulter)^60^,^61^. Cell staining was performed by CXCR5-FITC (clone: RF8B2, BD Pharmingen), CXCR3-PE (clone: 1C6, BD Pharmingen), CD4-PerCP-Cy5.5 (clone: OKT4, Biolegend), CD3-PE-Cy7 (clone: VCHT1, Biolegend), CCR6-APC (clone: 11A9, BD Pharmingen), and CD45RO-APC-Cy7 (clone: UCHL1, BD Pharmingen). The definitions of each subset in this study were as follows: CD3^+^CD4^+^CD45RO^+^ for memory CD4^+^ T cells, CD3^+^CD4^+^CD45RO^-^ for naive CD4^+^ T cells, CD3^+^CD4^+^CD45RO^+^CXCR5^+^ for follicular helper T cells (Tfh), CD3^+^CD4^+^CD45RO^+^CXCR5^−^CXCR3^+^CCR6^−^ for Th1, CD3^+^CD4^+^CD45RO^+^CXCR5^−^CXCR3^−^CCR6^+^ for Th17, and CD3^+^CD4^+^CD45RO^+^CXCR5^−^CXCR3^−^CCR6^−^ for non-Th1/Th17/Tfh. We followed standard immunophenotyping approach of human immune cells based on chemokine receptors, which is now becoming widely accepted^47^. The total count of sorted cells for the NGS analysis was listed in Table S2.

### Single-cell analysis

Single-cell whole transcriptome amplification was performed as an initial process for every single-cell analysis. We followed the original Kurimoto method^19^. Pre-amplified cDNA was used for the TCR repertoire and gene expression analysis. We focused on the beta chains of TCR because they generally have a more strict allelic exclusion than alpha chains. Detailed methods are described in the supplementary methods.

### NGS TCR repertoire analysis

We also focused on the beta chains of TCR in the NGS TCR repertoire analysis. We modified the 5′-RACE-based PCR strategy for the TCR region described previously^62^. Mapping to the TCR region, identification of the TRBV gene, extraction of CDR3 sequences, and error corrections were performed by MiTCR with default settings^18^. Detailed methods are described in the supplementary methods.

### Statistical analysis

Comparisons of numerical data between samples were analyzed by the unpaired Student’s t-test. p < 0.05 was considered significant. Spearman’s correlation coefficient was used to indicate the relationship between two ordered sets of numbers and was calculated by R (version3.0.2).

## Additional Information

**How to cite this article**: Ishigaki, K. *et al.* Quantitative and qualitative characterization of expanded CD4^+^ T cell clones in rheumatoid arthritis patients. *Sci. Rep.* **5**, 12937; doi: 10.1038/srep12937 (2015).

## Supplementary Material

Supplementary data
