# Supplementary material for: Quantitative and qualitative characterization of expanded CD4+ T cell clones in rheumatoid arthritis patients
Source: Sci Rep. 2015 Aug 6;5:12937. doi: 10.1038/srep12937 (PMC4542667; doi:10.1038/srep12937)
Supplement: Supplementary data [file srep12937-s1.pdf]

Title:

Quantitative and qualitative characterization of expanded CD4^{+} T cell clones in rheumatoid arthritis patients

Names of the authors:

Kazuyoshi Ishigaki[1], Hirofumi Shoda[1], Yuta Kochi[2], Tetsuro Yasui[3], Yuho Kadono[3], Sakae Tanaka[3], Keishi Fujio*[1] & Kazuhiko Yamamoto[1]

Affiliations:

1: Department of Allergy and Rheumatology, Graduate School of Medicine, the University of Tokyo, Tokyo, Japan.

2: Laboratory for Autoimmune Diseases, Center for Integrative Medical Sciences, RIKEN, Yokohama, Japan.

3: Department of Orthopaedic Surgery, Graduate School of Medicine, the University of Tokyo, Tokyo, Japan.

Corresponding author:

Keishi Fujio, M.D., Ph.D.

Department of Allergy and Rheumatology, Graduate School of Medicine, the University of Tokyo

7-3-1, Hongo, Bunkyo-ku, Tokyo, 113-0033, Japan

Tel: +81-3-3815-5411, Fax: +81-3-3815-5954, E-mail address: kfujio-tky@umin.ac.jp

Supplemental Materials and Methods

Content:

Supplementary methods (pages 2-4)

Supplementary results & figures (pages 5-6)

Supplementary tables (pages 7-9)

Supplementary references (page 10)

Supplementary methods

I, Single-cell analysis

Single-cell sorting

Memory $CD4^+$ T cells in the PB of RA1 and RA2 and in the synovium of RA1 were single-cell sorted by FACS (MoFlo XDP, Beckman Coulter).

Single-cell whole transcriptome amplification

Single-cell whole transcriptome amplification was performed as an initial process for every sample in the single-cell analysis. We followed the original Kurimoto method,[1] except that the reverse transcription duration was increased from 5 minutes to 50 minutes because this modification increased the success rate of TCR CDR3 sequencing. Pre-amplified cDNA was used for the TCR repertoire and gene expression analysis. Briefly, each single cell was directly sorted into 5 µL lysis buffer in the PCR tube, followed by cell lysis and heat denaturing. Reverse transcription was performed by SuperScript III (Invitrogen) and oligo-dT primer (V1_dT_24), followed by the exonuclease I (Takara) digestion of unreacted primers. Poly-A was added by TdT (Invitrogen). Each single-cell cDNA was split into 4 tubes to minimize the stochastic effects of amplifying small samples by PCR, and whole transcriptome amplification was performed by 20 cycles of PCR with oligo-dT primers (V1_dT_24 and V3_dT_24). The PCR products of each single cell (in 4 tubes) were mixed and purified using the QIAquick PCR purification kit (Qiagen). All primer sequences in this method section were shown in Table S4.

Single-cell TCR repertoire analysis

PCR strategy: We focused on the beta chains of TCR because they have generally a more strict allelic exclusion than alpha chains. We prepared forward primer sets (V_beta_common_2-24) to cover all of the functional TRBV as previously described[2] and also designed a reverse primer at TRBC (C_beta_332). We performed 50 cycles of multiplex PCR using pre-amplified single-cell cDNA as a template. PCR products were analyzed by electrophoresis on a 1.5% agarose gel. The fragment containing the target PCR product was cut out and purified using the QIAquick gel extraction kit (Qiagen).

Sequencing and mapping: Sequencing was performed by the Sanger method. Sequencing data was analyzed by IMGT/V-QUEST.[3]

Data analysis: The frequency of each clone was calculated by the number of cells with the same CDR3 sequence divided by the total number of sorted single cells. In the single-cell analysis, expanded $CD4^+$ T cell clones (ECs) were defined by the clones detected more than once. The most expanded memory $CD4^+$ T cell clones (mECs) were defined by the clones with the highest frequency in the single-cell analysis. Non-expanded $CD4^+$ T cell clones (NEC) were defined by the clones detected only once.

Single-cell quantitative PCR

We used pre-amplified single-cell cDNA as a template. Due to the limitation of the cDNA sample volume, we divided single cells into several groups according to the TCR sequences. Every single-cell cDNA of each group was mixed and quantitative PCR (qPCR) was performed for each pooled sample. qPCR was performed in the CFX Connect Real-Time PCR Detection System (BioRad) by using QuantiTect SYBR Green PCR Kits (Qiagen). The expression of the target

genes was calculated based on the $2^{-\text{delta Ct}}$ method, in which delta Ct = (Ct of the target gene - Ct of the internal control gene)[4]. All gene specific primers were listed in Table S4.

Single-cell RNA-seq

Library preparation and sequencing: We performed 9 cycles of PCR with oligo-dT primers (T7-V1 and V3_dT_24) using pre-amplified single-cell cDNA as a template and PCR products longer than 200bp were cut out on a 2% agarose gel and purified using the QIAquick gel extraction kit (Qiagen) as described in the original Kurimoto method.[1] We required an additional 30 cycles of PCR with the same conditions in order to obtain a sufficient amount of the PCR product for the NGS analysis. After the final PCR products were fragmented using the Covaris S2 system, they were ligated with index and adaptor sequences for the Ion Torrent platform. Sequencing was performed on Ion PGM using 318 v2 chips (Applied Biosystems).

Quality control, mapping, and data analysis: Raw sequencing data was trimmed and mapped with default settings on the CLC Genomics Workbench v.7.5.[5] The Ensembl gene model (GRCh37.72) was used. Gene expression levels were calculated by reads per kilobase of the exon model per million mapped reads (RPKM) values[6]. RPKM values were log-transformed ($\log_2$ (RPKM+1)) and normalized by the quantile method. These normalized RPKM values were used as transcriptome data for every analysis of RNA-seq.

Gene set enrichment analysis: Regarding the analysis of differentially expressed gene sets between 2 groups, a gene set enrichment analysis was performed as previously reported.[7] The number of permutations was set at 1000 and the false discovery rate (FDR) cut-off was set at 0.05.

II, NGS TCR repertoire analysis

Library preparation and sequencing: We also focused on the beta chain of TCR in the NGS TCR repertoire analysis. We modified the 5'-RACE-based PCR strategy of the TCR region described previously.[8] Briefly, total RNA was purified using the RNeasy Micro kit (Qiagen) and reverse transcribed with SuperScript III (Invitrogen) and gene-specific primers of the TCR constant region (hTCR-CA-R2.2, hTCR-CB1-R3.2 and hTCR-CB2-R3). Poly-G was added on the 5' end of each cDNA sample and they were split into four tubes to minimize the stochastic effects of PCR. In the 1st round of PCR, touch-down PCR was performed by the oligo-dC forward primer (Oligo dc-adaptor2) and reverse primers in the TCR constant region (hTCR-CA-R7 and hTCR-CB1-R9) using the same conditions as the original method,[8] except that the final number of amplification cycles was reduced to 16 cycles. After the 1st PCR products of each sample (in 4 tubes) were mixed together, they were diluted 20-fold and used as a template in the subsequent PCR process. The 2nd round of PCR was performed using a forward primer with an adaptor region (AP2) and reverse primer in the TCR constant region of the beta chain (TRBC_HTS_3), using the following conditions: 1) 96°C for 2 minutes, 2) 18 cycles of 96°C for 15 seconds, 63°C for 30 seconds, and 72°C for 1 minute, and 3) 72°C for 3 minutes. The 2nd PCR products were purified using the QIAquick PCR purification kit (Qiagen). We used high-fidelity polymerase (PrimeStarGXL, Takara) in both rounds of PCR. After the final PCR products were fragmented by the Covaris S2 system, they were ligated with index and adaptor sequences for the Ion Torrent platform. Sequencing was performed on Ion PGM using 318 v2 chips (Applied Biosystems).

NGS TCR repertoire data analysis

Quality control and mapping: Quality control of raw sequencing data was performed on the CLC Genomics Workbench v.7.5.[5] After adaptor removal, further trimming was performed using the following settings: quality=0.01, no ambiguous nucleotide was allowed and reads below the length = 50bp were discarded. Mapping to the TCR region, the identification of TRBV and TRBJ genes, the extraction of CDR3 sequences, and error corrections were performed with default settings by MiTCR.[9] Functional CDR3 sequences were used for the TCR repertoire analysis.

Read depth control: As discussed in Fig. S1, we set the target total read count for each sample to be more than 100,000. However, the number of reads with functional CDR3 slightly varied between samples. In order to cancel the potential impacts of the read depth on the TCR repertoire analysis, functional CDR3 sequences were shuffled and an equal number of sequences (set as the minimum number within samples) were randomly extracted in each data set (Fig. 2 and 3).

Frequency calculation: The frequency of each clone was calculated by the number of reads of each clone divided by the total number of reads with functional CDR3 sequences. In the NGS analysis, ECs were defined by clones detected more frequently than 0.1% or 0.2% (Fig. 2). In order to assess the distribution of target clones within different subsets of $CD4^+$ T cells (Fig. 3), frequencies assessed by the TCR repertoire analysis were multiplied by the frequency of each $CD4^+$ T cell subset within all $CD4^+$ T cells assessed by FACS in order to correct for the subset size.

Supplementary results & figures

Validness of error corrections and estimation of optimal read depths in the NGS TCR repertoire analysis (Fig. S1)

The advantage of the NGS TCR repertoire analysis was the high throughput read and relative simpleness. However, it is limited by the high error rate and PCR bias during the library preparation process. We first examined the impact of error correction pipeline MiTCR[9] and estimated optimal read depths. We randomly re-sampled several pairs of datasets with the same number of reads from 2 NGS sequence data (RA1 and RA2), and calculated the consistency rate of CDR3 sequences between pairs with or without error corrections (consistency rate = total number of consistent clones between set 1 and set 2 / total number of unique clones in set 1 and set 2). The chances of detecting consistent reads between pairs generally become larger as the depth of the sequence became deeper. However, reads originating from sequencing errors increased linearly as the depth of the sequence became deeper and the majority of error-derived reads were counted as a "unique clone" by the definition due to the random nature of sequencing errors. Therefore, we observed that the consistency rate curve had a peak and decline phase without any error correction (Fig. S1 left). When sequencing errors were corrected with default settings by MiTCR,[9] the consistent rate curve had an upward-sloping shape and finally became a plateau as the samples were exhaustively sequenced (Fig. S1 right). These results verified the robustness of the error correction process and indicated 100,000 reads could cover the majority of clones in each sample.

Validation of the NGS TCR repertoire analysis by FACS and single-cell analyses (Fig. S2)

We performed a validation experiment to confirm the correctness of our NGS TCR repertoire analysis pipelines by FACS and single-cell TCR repertoire analyses with Sanger sequencing, neither of which were affected by PCR bias. Comparisons of TRBV usage data between FACS and NGS TCR repertoire analyses revealed a strong correlation (Fig. S2A and S2B). Evaluations of the frequency of each clone also showed a significant correlation between single-cell and NGS TCR repertoire analysis (Fig. S2C), although the correlation coefficient was smaller than that for TRBV usage data. We speculated that the wider dynamic range of the NGS TCR repertoire analysis for low-frequency clones hampered a strong correlation. In summary, we verified that our NGS TCR repertoire analysis pipeline had sufficient correctness.

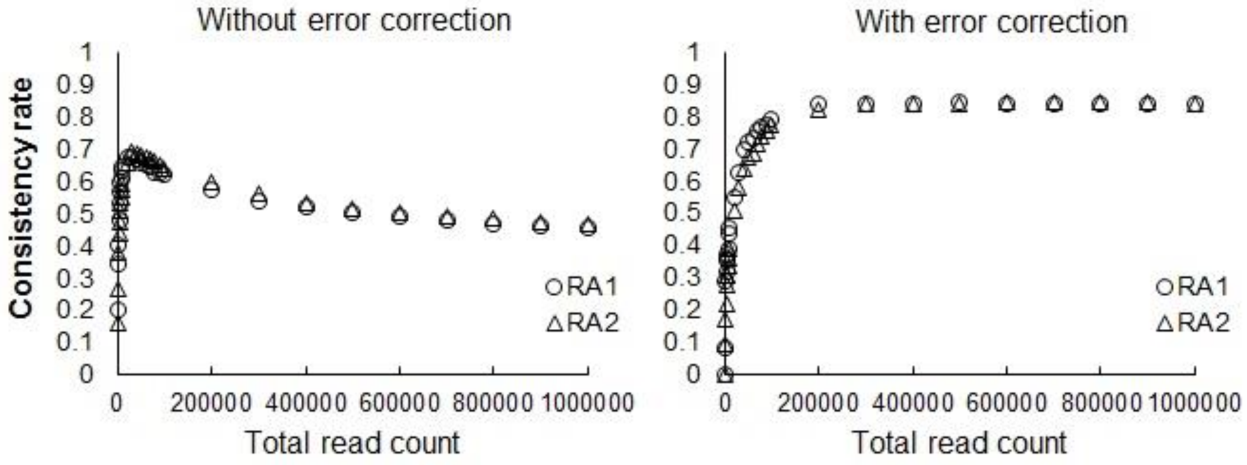

Supplemental figure 1 Validity of error corrections and estimation of optimal read depths in the NGS TCR repertoire analysis

One equal-sized pair of data were randomly re-sampled (each read count was shown in the X-axis) from whole sequence data obtained by the NGS TCR repertoire analysis of memory $CD4^{+}$ T cells from RA1 and RA2. The consistency rate of the CDR3 sequence between pairs with or without error corrections was shown (consistency rate = total number of consistent clones between set 1 and set 2 / total number of unique clones in set 1 and set 2).

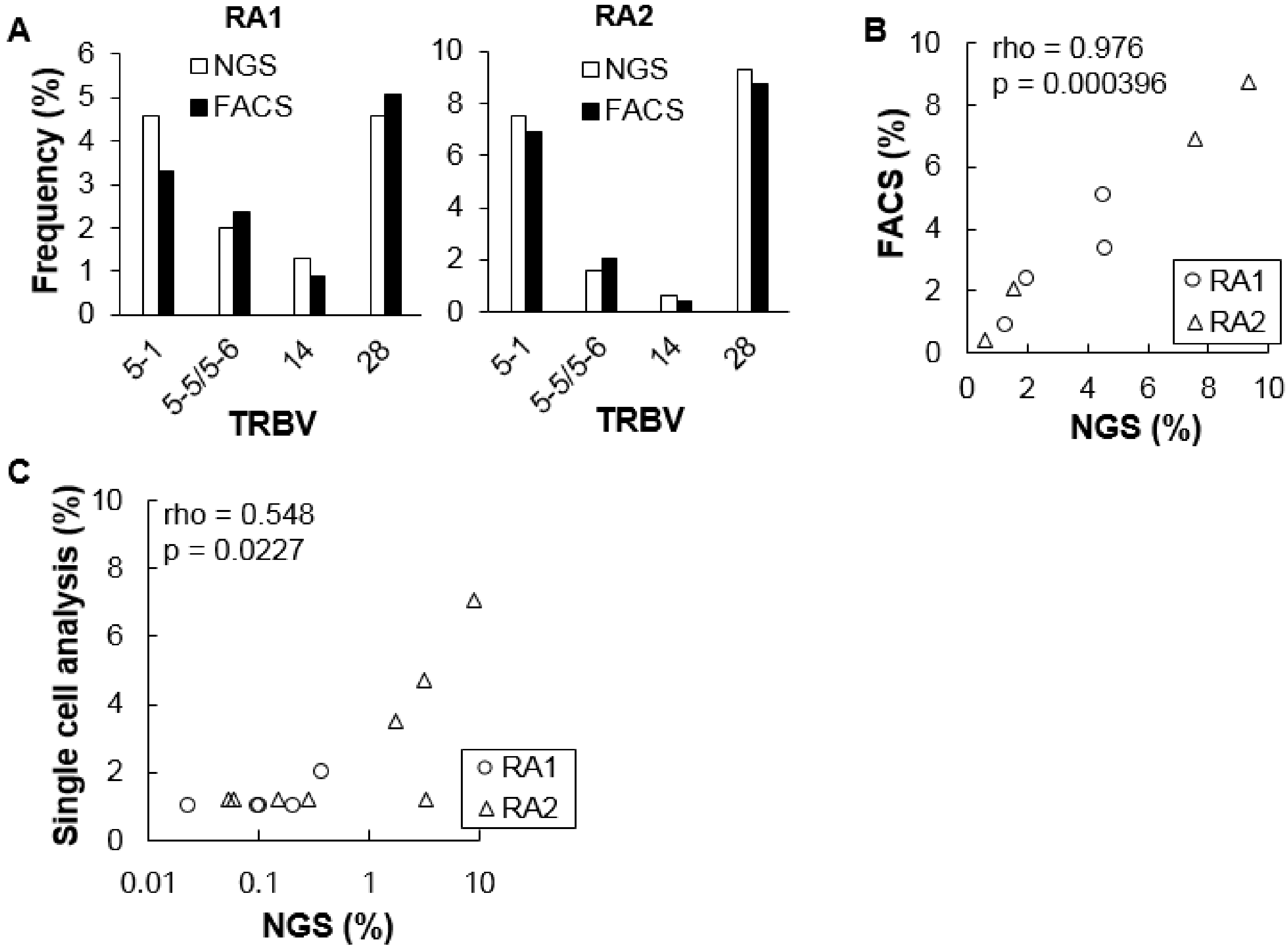

Supplemental figure 2 Validation of the NGS TCR repertoire analysis by FACS and single-cell analyses

A, B, TRBV usage data was compared between FACS and NGS TCR repertoire analyses. Each dot (B) indicates one TRBV usage frequency. **C,** The frequency of each clone detected both in single-cell and NGS TCR repertoire analysis were compared. Each dot indicates one clone. Spearman’s correlation coefficient was used (B, C).

Supplementary tables

	RA1	RA2	RA3	RA4	RA5
**Age / Sex**	**60**	**69**	**61**	**58**	**53**
**Sex**	**F**	**F**	**F**	**F**	**M**
**Disease duration (year)**	**37**	**31**	**18**	**25**	**0.2**
**Tender joint count**	**5**	**ND**	**8**	**1**	**22**
**Swollen joint count**	**6**	**ND**	**2**	**3**	**20**
**PtGA [/100mm]**	**60**	**ND**	**27**	**48**	**60**
**EGA [/100mm]**	**60**	**ND**	**35**	**36**	**80**
**CRP [mg/dL]**	**3.84**	**0.34**	**0.32**	**0.32**	**8.34**
**ESR [mm/1hr]**	**102**	**ND**	**30**	**28**	**83**
**RF [U/mL]**	**248**	**149**	**24**	**41**	**410**
**Anti-CCP ab [U/mL]**	**16.6**	**100**	**194**	**ND**	**361**
**HLA-DRB1**	**0405/0101**	**0405/0405**	**0405/1502**	**ND**	**0405/0803**
**DAS28 (CRP)**	**5.18**	**ND**	**3.93**	**3.19**	**7.28**
**DAS28 (ESR)**	**6.02**	**ND**	**4.74**	**4.05**	**7.81**
**SDAI**	**17**	**ND**	**12.7**	**8.8**	**48**
**Treatment**	**ETN 50mg/W**<br>**mPSL 4mg**	**MTX 6mg/W**	**ETN 50mg/W**<br>**MTX 12.5mg/W**<br>**PSL 4mg**	**ETN 25mg/W**<br>**MTX 8mg/W**	**No treatment**
**Operation**	**Elbow Synovectomy**	**ND**	**ND**	**ND**	**ND**

Supplemental table 1 Basic patient information

PtGA: patient's global assessment, EGA: examiner's global assessment, ETN: etanercept, mPSL: methylprednisolone, PSL: prednisolone, MTX: methotrexate

	RA1	RA2	RA3	RA4	RA5	HC1	HC2	HC3	HC4	HC5
**Memory CD4$^+$ T cells**	10	10	-	10	18	10	10	67	74	42
**Naïve CD4$^+$ T cells**	10	10	-	10	20	10	10	77	64	60
**Tfh**	10	9	7	-	-	-	-	-	-	-
**Th1**	5	7	4	-	-	-	-	-	-	-
**Th17**	6	4	3	-	-	-	-	-	-	-
**Non-Th1/Th17/Tfh**	10	10	6	-	-	-	-	-	-	-

Supplemental table 2 Sorted cell counts (x 10^4 cells)

Clone ID	TRBV	TRBJ	CDR3
**C1.1**	**7-2**	**1-5**	**ASSSEANQPQH**
**C1.2**	**12-3**	**2-3**	**ASSLEGSGTDTQY**
**C2.1**	**16**	**1-2**	**ASSQAGIGYT**

**C2.2**	**18**	**1-4**	**ASSPGQGEKLF**
**C2.3**	**6-5**	**2-1**	**ASSYRRPSSYNEQF**
**C2.4**	**28**	**1-1**	**ASSLPQGTTEAF**
**C2.5**	**5-1**	**1-2**	**ASSLDGKGYT**

Supplemental table 3 TRBV gene, TRBJ gene, and CDR3 amino acid sequences of the major memory-ECs in RA1 and RA2

Name	Sequence(5'-3')
**V1_dT_24**	**ATATGGATCCGGCGCGCCGTCGACTTTTTTTTTTTTTTTTTTTTTTTT**
**V3_dT_24**	**ATATCTCGAGGGCGCGCCGGATCCTTTTTTTTTTTTTTTTTTTTTTTT**
**T7-V1**	**GGCCAGTGAATTGTAATACGACTCACTATAGGGAGGCGGATATGGAT CCGGCGCGCCGTCGAC**
**V_beta_common_2**	**AACTATGTTTTGGTATCGTCA**
**V_beta_common_4**	**CACGATGTTCTGGTACCGTCAGCA**
**V_beta_common_5/1**	**CAGTGTGTCCTGGTACCAACAG**
**V_beta_common_6a11**	**AACCCTTTATTGGTACCGACA**
**V_beta_common_6a25**	**ATCCCTTTTTTGGTACCAACAG**
**V_beta_common_6c**	**AACCCTTTATTGGTATCAACAG**
**V_beta_common_7**	**CGCTATGTATTGGTACAAGCA**
**V_beta_common_8a**	**CTCCCGTTTTCTGGTACAGACAGAC**
**V_beta_common_9**	**CGCTATGTATTGGTATAAACAG**
**V_beta_common_10**	**TTATGTTTACTGGTATCGTAAGAAGC**
**V_beta_common_11**	**CAAAATGTACTGGTATCAACAA**
**V_beta_common_12a**	**ATACATGTACTGGTATCGACAAGAC**
**V_beta_common_13b**	**GGCCATGTACTGGTATAGACAAG**
**V_beta_common_13c**	**GTATATGTCCTGGTATCGACAAGA**
**V_beta_common_16**	**TAACCTTTATTGGTATCGACGTGT**
**V_beta_common_17**	**GGCCATGTACTGGTACCGACA**
**V_beta_common_18**	**TCATGTTTACTGGTATCGGCAG**
**V_beta_common_19**	**TTATGTTTATTGGTATCAACAGAATCA**
**V_beta_common_20**	**CAACCTATACTGGTACCGACA**
**V_beta_common_21**	**TACCCTTTACTGGTACCGGCAG**
**V_beta_common_22**	**ATACTTCTATTGGTACAGACAAATCT**
**V_beta_common_23**	**CACGGTCTACTGGTACCAGCA**
**V_beta_common_24**	**CGTCATGTACTGGTACCAGCA**
**C_beta_332**	**TGGGTCCACTCGTCATTCTCC**
**hTCR-CA-R2.2**	**TCAGGCAGTGACAAGCAGCAATAAGGGAAC**
**hTCR-CB1-R3.2**	**CCATGACGGGTTAGAAGCTCCTAACTCC**
**hTCR-CB2-R3**	**CTG GGATGGTTTTGGAGCTA**
**Oligo dc-adaptor2**	**ACAGCAGGTCAGTCAAGCAGTAGCAGCAGTTCGATAACATCCCCCCC**

	CCCCCDN
hTCR-CA-R7	TCTCAGCTGGACCACAGCCGCAGCGTCA
hTCR-CB1-R9	VVGRAATCCTTTCTCTTGACCATGGCCATCA
AP2	AGCAGTAGCAGCAGTTCGATAA
TRBC_HTS_3	CGACCTCGGGTGGGAACA
ACTB_Fw	TCCTGTGGCATCCACGAAACT
ACTB_Rv	GAAGCATTTGCGGTGGACGAT
TBX21_Fw	TAATCCCTGATCCAAAAAGA
TBX21_Rv	CACAACCACCACAAACAATA
FoxP3_Fw	TATCTCACGCATATGCACAC
FoxP3_Rv	CTTGGATCCCAAATAAATGA
GATA3_Fw	AGGGTCTCTAGTGCTGTGAA
GATA3_Rv	GTGAAAGGAAACAAAACTGG
RORC_Fw	AGTCGGAAGGCAAGATCAGA
RORC_Rv	CAAGAGAGGTTCTGGGCAAG
EOMES_Fw	GCTTCTGAAAGAGGGCTGTG
EOMES_Rv	CAATCCTGGACTAGGGCAAA
CXCR4_Fw	AACATTCCAGAGCGTGTAGT
CXCR4_Rv	CTATACCACTTTGGGCTTTG
CCR5_Fw	ATTTGGGGGATGGCTAAAAC
CCR5_Rv	CTGACAGCCACAATGCTCAC
CD28_Fw	CAGCAGTACTTGGGTGCTGA
CD28_Rv	TATTTGCCACTGCCATTTCA
GZMB_Fw	GGAGGCCCTCTTGTGTGTAA
GZMB_Rv	ATTACAGCGGGGGCTTAGTT
CD5_Fw	CATGCTGGGAATCCAGAAAC
CD5_Rv	AGTAGGCCATGGTTGCTTTG
KLRK1_Fw	CAGGGGATCAGTGAAGGAAG
KLRK1_Rv	CCCATTAAAAGTGGCAGCAT
IFNG_Fw	AATTAGGCAAGGCTATGTGA
IFNG_Rv	ATATTTTCAAACCGGCAGTA
IL17_Fw	AATGAAGACATGGTCCTACG
IL17_Rv	GAGAATCACATGAGGTGACA
TNF_Fw	CTGACATCTGGAATCTGGAG
TNF_Rv	CTAAGGTCCACTTGTGTCAA

Supplemental table 4 Primer sequences

Supplementary references

1 Kurimoto, K., Yabuta, Y., Ohinata, Y. & Saitou, M. Global single-cell cDNA amplification to provide a template for representative high-density oligonucleotide microarray analysis. *Nat Protoc* **2**, 739-752, doi:10.1038/nprot.2007.79 (2007).

2 van Dongen, J. J. *et al.* Design and standardization of PCR primers and protocols for detection of clonal immunoglobulin and T-cell receptor gene recombinations in suspect lymphoproliferations: report of the BIOMED-2 Concerted Action BMH4-CT98-3936. *Leukemia* **17**, 2257-2317, doi:10.1038/sj.leu.2403202 (2003).

3 Brochet, X., Lefranc, M. P. & Giudicelli, V. IMGT/V-QUEST: the highly customized and integrated system for IG and TR standardized V-J and V-D-J sequence analysis. *Nucleic Acids Res* **36**, W503-508, doi:10.1093/nar/gkn316 (2008).

4 Schmittgen, T. D. & Livak, K. J. Analyzing real-time PCR data by the comparative C(T) method. *Nat Protoc* **3**, 1101-1108 (2008).

5 Tripathi, A. K. *et al.* Transcriptomic dissection of myogenic differentiation signature in caprine by RNA-Seq. *Mech Dev* **132**, 79-92, doi:10.1016/j.mod.2014.01.001 (2014).

6 Mortazavi, A., Williams, B. A., McCue, K., Schaeffer, L. & Wold, B. Mapping and quantifying mammalian transcriptomes by RNA-Seq. *Nat Methods* **5**, 621-628, doi:10.1038/nmeth.1226 (2008).

7 Subramanian, A. *et al.* Gene set enrichment analysis: a knowledge-based approach for interpreting genome-wide expression profiles. *Proc Natl Acad Sci U S A* **102**, 15545-15550, doi:10.1073/pnas.0506580102 (2005).

8 Sun, X. *et al.* Unbiased analysis of TCRα/β chains at the single-cell level in human CD8+ T-cell subsets. *PLoS One* **7**, e40386, doi:10.1371/journal.pone.0040386 (2012).

9 Bolotin, D. A. *et al.* MiTCR: software for T-cell receptor sequencing data analysis. *Nat Methods* **10**, 813-814, doi:10.1038/nmeth.2555 (2013).
